# Potassium Intake and the Prevalence of Metabolic Syndrome: The Korean National Health and Nutrition Examination Survey 2008–2010

**DOI:** 10.1371/journal.pone.0055106

**Published:** 2013-01-25

**Authors:** Hajeong Lee, Jeonghwan Lee, Seung-sik Hwang, Sejoong Kim, Ho Jun Chin, Jin Suk Han, Nam Ju Heo

**Affiliations:** 1 Department of Internal Medicine, Seoul National University Hospital, Seoul, Korea; 2 Department of Social and Preventive Medicine, Inha University School of Medicine, Incheon, Korea; 3 Department of Internal Medicine, Seoul National University Bundang Hospital, Seongnam, Korea; 4 Department of Internal Medicine, Healthcare System Gangnam Center, Seoul National University Hospital, Seoul, Korea; Foundation for Liver Research, United Kingdom

## Abstract

Lower potassium intake is considered to be correlated with diabetes incidence. However, few studies have investigated the effect of potassium intake on metabolic syndrome (MetS). Data was taken from the Korean National Health and Nutritional Examination Survey (2008–2010) using weighted adjustment. MetS was defined as per the revised National Cholesterol Education Program criteria. Homeostasis model assessment indices were calculated to diagnosis insulin resistance (IR). A total of 16,637 participants (44±0.25 years) were included. Women ingested lower amounts of potassium (2.71±0.02 g/day) than men (3.45±0.03 g/day). A curvilinear association between potassium intake and MetS prevalence was found among women. Women with less than the Adequate Intake (4.7 g/day) of potassium had an 11% risk reduction for MetS (adjusted odds ratio [OR], 0.89; 95% confidence interval [CI], 0.82–0.96; *P = *0.004) and a 10% risk reduction for IR (OR, 0.90; 95% CI, 0.82–0.99; *P = *0.026) for every 1 g/day potassium increase. Compared with the reference group (3.5–4.5 g/day), potassium intake was inversely associated with an increased risk of MetS (1.5–2.5 g/day; OR, 1.29; 95% CI, 1.02–1.63; *P = *0.035; <1.5 g/day; OR, 1.40; 95% CI, 1.06–1.85; *P = *0.017) and IR (<1.5 g/day; OR, 1.36; 95% CI, 1.05–1.76; *P = *0.021). This relationship was more prominent in postmenopausal women, but not observed among men. Higher potassium intake is significantly associated with a lower MetS prevalence in women, and IR is believed to be connected.

## Introduction

To date, substantial evidence showed that dietary deficiency in potassium result in increased blood pressure [1]–[3], salt sensitivity [4], and consequently elevates the risk of cardiovascular morbidity [5], [6] and mortality [7]. Recent population-based studies have also demonstrated that lower dietary potassium is associated with new onset diabetes in African-Americans [8]. In addition, both dietary and serum potassium levels have been established as a novel risk factor for thiazide induced diabetes [9], [10]. However, limited data is available regarding the impact of potassium on metabolic syndrome (MetS) in the general population.

MetS is a cluster of metabolic risk factors including high blood pressure, hyperglycemia, central obesity, hypertriglyceridemia and low high density lipoprotein (HDL) cholesterol levels. Through these metabolic abnormalities, MetS is associated with the development of type 2 diabetes [11], elevated cardiovascular morbidity, and all-cause mortality [12]. Similar to that in the US population [13], the prevalence of MetS is rapidly increasing in Korea, from 24.9% in 1998, 29.2% in 2001, and 30.4% in 2005 to 31.3% in 2007 [14]. These increases in MetS prevalence may increase subsequent health care burdens associated with diabetes or cardiovascular disease. Several recent studies have reported on the association of reduced MetS risk with certain dietary factors, including higher intakes of crude fiber, whole grains, fruits, and vegetables [15]. Although such foods could also be rich in potassium, studies evaluating the relationship between dietary potassium and MetS have not been reported. A recent experimental study demonstrated that thiazide-induced potassium depletion with normo-kalemia exacerbated hyperglycemia and insulin resistance (IR**)** in the fructose induced model of MetS, all of which were ameliorated by potassium supplements [16]. Such results suggest that higher potassium intake may alleviate MetS risk. However, evidence on the protective effect of potassium on MetS is scarce in humans. Therefore, this study was designed to analyze the association between dietary potassium intake and MetS. We hypothesized that lower dietary potassium intake would correlate with a higher risk of MetS in the general population.

## Materials and Methods

### Ethics Statement

This investigation was conducted according to the principles expressed in the Declaration of Helsinki. The participants of KNHANES engaged voluntarily. The KNHANES has been performed since 1998 and received ethical approval by Institutional Review Board of Korea Center for Disease Control & Prevention (IRB No: 2008-04EXP-01-C, 2009-01CON-03-2C, 2010-02CON-21-C).

### Study Participants

The data analyzed in this study were obtained from the Korean National Health and Nutritional Examination Survey (KNHANES) performed between 2008 and 2010, which included individuals from households across 600 national districts. Using a rolling survey sampling method, the extracted participants were similarly distributed each year. From a total of 29,235 participants, those who completed Health Behavior survey, a Health Examination Survey, and a Nutrition Survey were included in this investigation. Individuals were excluded if they were <19 years of age (n = 8,054), did not complete a blood pressure check (n = 1,127) or waist circumference measurement (n = 1,260), did not provide laboratory data (n = 1,486), advanced renal dysfunction with an estimated glomerular filtration rate lower than 30 mL/min/1.73 m^2^ (n = 26), did not participate in the Nutritional Survey (n = 2,621), did not have their weight measured (n = 4,442).

### Estimating Usual Potassium Intake

Nutritional information was collected using a 24-h dietary recall and food frequency questionnaire that included 63 food items, and was administered by a trained dietary interviewer from the Korea Health Industry Development Institute. The 24-h recall was based on a single weekday’s food intake and the nutrients were quantified using the Database Management System (Korea Health Industry Development Institute). For a single 24-h recall, supplementary tools such as food models and two-dimensional food volumes and containers were used. Dietary nutrient intakes were assessed by the provided energy and 11 nutrients based on the dietary reference intakes for Koreans. In addition, a quantitative food frequency diet survey was conducted, covering over 60,000 items to examine participants’ food intake history over the preceding year. From these data, we selected diet variables including energy intake, carbohydrate intake as a percentage of energy, and crude fiber, all of which were derived from the same quantitative food frequency survey that was used to calculate the dietary potassium intake. It was validated in Korea and published by the Rural Department Administration, Korean Ministry of Food, Agriculture, Forestry and Fisheries [17]. Vegetable intake was defined as having 2 or more vegetables once or more per day. Fruit intake was defined as the consumption of at least one fruits, at least once per day.

### Measurements of Metabolic Risk Factors

Assessed demographic characteristics included age; gender; anthropometric measurement; health-related behaviors; and socioeconomic factors including education level, income, and area of residence. Blood pressure was manually measured by trained examiner, 3 times, at 30-s intervals, using the subject’s right arm after a minimum of 5 min of rest in a seated position. The average of the second and third readings was considered as the final blood pressure. Height and weight were measured with the participant wearing light clothing and no shoes. Body mass index (BMI) was computed as weight (kg) divided by the height (m) squared. Waist circumference was measured by placing a tape measure at the midpoint between the inferior margin of the last rib and the iliac crest in the mid-axillary plane, while the participant maintained a full expiratory position.

Information on health-related behaviors such as smoking status (non-smoker, ex-smoker, or current smoker), alcohol intake (less than once per month, less than once per week, or more than twice per week), and regular physical activity of moderate intensity (less or more than 3 times per week) was obtained from the health questionnaire. Moderate-intensity activities were defined as those lasting at least 10 min and causing a slight increase in the individual’s heart rate compared with sedentary activities; table tennis, swimming, yoga and badminton were included as moderate-intensity activities, but walking was excluded. Education was graded as none or elementary school, middle school, high school, university or higher. Economic status was represented by monthly individual income divided into quartiles. An urban residence was defined as the central districts in 7 metropolitan areas. Information on menstruation state and hormone replacement therapy was also acquired from the survey.

During the survey, blood samples were collected after a 12-h overnight fast, and were properly processed, immediately refrigerated and transported in cold storage to a central laboratory within 24 h. Fasting plasma glucose, and lipid profile including triglyceride, and HDL-cholesterol levels were determined by enzymatic method and a Hitachi Automatic Analyzer 7600 (Hitachi, Tokyo, Japan). Insulin levels were measured using a gamma counter (1470 Wizard; Perkin-Elmer, Turtu, Finland) with an immunoradiometric assay (Biosources, Nivelles, Belgium). The intra- and inter-assay coefficients of variation were 1.6–2.2% and 6.1–6.5%, respectively. To estimate IR, homeostasis model assessment (HOMA) was used, according to the following equation: HOMA-IR = fasting insulin (µU/mL) × fasting glucose (mg/dL)/405 [18]. There was no report of standard of IR at HOMA index in Koreans. Therefore, we selected the population-specific 75^th^ percentile of HOMA-IR as the probability of IR [19].

### Definitions

MetS components that were examined included high blood pressure, hyperglycemia, central obesity, low HDL-cholesterol and hypertriglyceridemia, according to the revised NECP-ATP III guidelines. High blood pressure was defined as systolic blood pressure (SBP) ≥130 mmHg or diastolic blood pressure (DBP) ≥85 mmHg or self-reported treatment with antihypertensive drugs. Hyperglycemia was defined as fasting plasma glucose level ≥100 mg/dL or self-reported ongoing treatment with an oral hypoglycemic agent or insulin. Central obesity was adjusted for Korean as waist circumference ≥90 cm for men or ≥80 cm for women [20], low HDL cholesterol and high triglyceride levels were defined as HDL-cholesterol levels <40 mg/dL for men or <50 mg/dL for women, and triglyceride levels ≥150 mg/dL. Diagnosis of MetS was based on the presence of 3 or more of the 5 components. Hypertension was identified in individuals who met at least one of the following 3 criteria: physician diagnosis of hypertension, self-report antihypertensive drug therapy, and SBP≥140 mmHg or DBP≥90 mmHg. Diabetes was diagnosed in subjects with a fasting plasma glucose ≥126 mg/dL or those patients who were identified in the health interview survey as actively using an oral hypoglycemic agent or insulin.

### Data Analysis

All analyses were performed separately for men and women because there were significant differences in dietary behaviors and definitions of MetS, and there were interactions between potassium intake and sex. Survey analyses were conducted in consideration of weighting to reflect complex survey sampling, unequal probabilities of selection, non-response adjustments and to produce unbiased estimates generalizable to the Korean population. Data are presented as means with SE for continuous variables and percentages for categorical variables. Dose-response associations were initially explored between potassium intake and MetS/IR by fitting a restricted cubic spline function. Women with potassium intake of 3.5–4.5 g/day were selected as the reference group because this was the range associated with the lowest risk of MetS in the spline plot. Additionally, the reference value of 4.7 g/day were used because it is the recommended Adequate Intake level for potassium in the US Dietary Guidelines [21]. Because menstruation status influences the cardiovascular risk in women, the association between potassium and MetS was also analyzed according to menstruation status.

ANOVA and *χ^2^* test were used to determine whether there were differences in the risks for MetS/IR among the potassium groups. Potential confounding variables identified *a priori* included age, BMI, alcohol, smoking, exercise, education, income, area of residence, frequencies of vegetable and fruit intake, energy intake, carbohydrate energy ratio, and fiber intake. Variables that showed significant associations (*P*<0.10) in the univariate weighted logistic regression analysis or were of considerable theoretical relevance were entered into the multivariate weighted logistic regression analysis. Moreover, the adjusted cubic spline curve, adjusted for the aforementioned confounding variables, was fit to further characterize the nature of the relationship between potassium intake and MetS. Five knots were chosen because this number produced a curve that appeared adequately smooth. Because the most accepted hypothesis to describe the pathophysiology of the MetS is IR, weighted logistic regression analyses on HOMA-IR were performed. All analyses were performed using the STATA software version 12.0 (Stata, College Station, TX, USA). All reported probabilities were two-sided, with a *P*-value <0.05 considered to be statistically significant.

## Results

### Baseline Characteristics

After taking into account the described exclusions, a total of 6,726 men and 9,911 women were included in the final analyses. The participants demographic information is summarized in Table 1. In the overall population, the mean potassium intake ± SE was 3.08±0.02 g/day and was definitely lower among women (2.71±0.02 g/day), than among men (3.45±0.03 g/day). The prevalence of diabetes mellitus and hypertension were higher in men than in women; however, the prevalence of MetS was not different between genders. Among the 5 components of MetS, women exhibited a higher prevalence of central obesity and low HDL-cholesterol levels than did men. Women tended to smoke less, drink less alcohol, and to be less educated. Although women consumed fewer calories and more fruit, they also consumed fewer vegetables and higher proportions of carbohydrates than men. Approximately 46.5% of women (n = 4,583) were menopausal and 15.8% (n = 725) of the menopausal women were receiving hormone replacement therapy.

**Table 1:** Demographic and clinical characteristics of overall study population.

Variables	Total	Men	Women
N	16,637	6726	9911
Age (years)	44.5±0.25	43.7±0.30	45.3±0.27
BMI (kg/m^2^)	23.6±0.04	24.1±0.05	23.2±0.05
Potassium intake (g/day)	3.08±0.02	3.45±0.03	2.71±0.02
Diabetes (%)	8.3 (7.8–8.8)	9.0 (8.3–9.8)	7.6 (6.9–8.2)
Hypertension (%)	26.8 (19.7–21.3)	30.6 (29.3–32.0)	22.9 (21.8–24.0)
MS (%)	20.5 (19.7–21.3)	20.7 (19.6–21.9)	20.3 (19.3–21.3)
Number of MS components	1.43 (1.40–1.45)	1.49 (1.45–1.53)	1.37 (1.33–1.40)
High blood pressure (%)	43.8 (42.5–45.0)	53.7 (51.9–55.3)	34.0 (32.6–35.4)
Hyperglycemia (%)	11.4 (10.8–12.0)	13.6 (12.6–14.6)	9.2 (8.5–9.9)
Central obesity (%)	32.1 (31.0–33.3)	25.0 (23.6–26.4)	39.2 (37.8–40.7)
Low HDL (%)	27.5 (26.6–28.5)	20.2 (19.0–21.5)	34.8 (33.6–36.0)
Hypertriglyceridemia (%)	28.0 (27.1–28.9)	36.6 (35.1–38.0)	19.5 (18.5–20.4)
Smoking (%): Never	53.6 (52.7–54.5)	19.9 (18.7–21.1)	87.0 (86.0–88.0)
Ex-smoker	6.9 (6.4–7.4)	11.4 (10.5–12.5)	2.4 (1.9–2.9)
Current smoker	39.7 (38.6–40.5)	68.7 (67.3–70.1)	10.7 (9.8–11.6)
Alcohol (%) <1/month	41.1 (40.1–42.2)	24.2 (23.0–25.5)	57.9 (56.7–59.2)
1/mon∼1/week	35.7 (34.7–36.6)	39.2 (37.8–40.7)	32.1 (30.9–33.2)
≥2/week	23.2 (22.4–24.1)	36.5 (35.1–38.0)	10.0 (9.2–10.9)
Exercise (%)	25.9 (24.9–27.0)	26.9 (25.6–28.3)	24.9 (23.7–26.3)
Education (%) <middle	18.7 (17.7–19.8)	12.3 (11.3–13.3)	25.2 (23.8–26.5)
Middle school	10.4 (9.8–11.0)	10.8 (9.9–11.7)	10.1 (9.4–10.8)
High school	39.6 (38.4–40.8)	41.6 (40.0–43.2)	37.6 (36.3–39.1)
≥University	31.2 (29.9–32.6)	35.4 (33.6–37.1)	27.2 (25.8–28.6)
Income (%): 1Q	24.8 (23.6–26.2)	25.0 (23.4–26.7)	24.6 (23.3–26.1)
2Q	25.1 (24.1–26.2)	25.6 (24.2–27.0)	24.7 (23.5–25.9)
3Q	25.0 (24.0–26.0)	24.3 (23.0–25.6)	25.6 (24.5–26.8)
4Q	25.1 (23.7–26.6)	25.2 (23.5–26.9)	25.0 (23.5–26.7)
Urban residence (%)	47.6 (45.4–49.8)	47.1 (44.6–49.5)	48.2 (46.0–50.4)
Vegetable intake (≥2/day, %)	41.0 (39.8–42.3)	44.1 (43.0–46.3)	37.4 (36.0–38.9)
Fruit intake (≥1/day, %)	7.0 (6.4–7.7)	5.7 (5.0–6.5)	8.4 (7.6–9.2)
Fiber (g/day)	7.6±0.07	8.2±0.09	6.9±0.08
Energy intake (Kcal/day)	1984±10.2	2328±14.9	1644±9.1
Carbohydrate energy ratio (%)	65.9±0.17	62.8±0.24	69.1±0.18

Data are presented with survey means ± standard errors for continuous variables and weighted percentages (95% confidence interval) for categorical variables.

Abbreviations: BMI, Body mass index; MS, metabolic syndrome; HRT, hormone replacement therapy; HDL, high density lipoprotein.

### Potassium Intake and MetS

**Figure 1:**
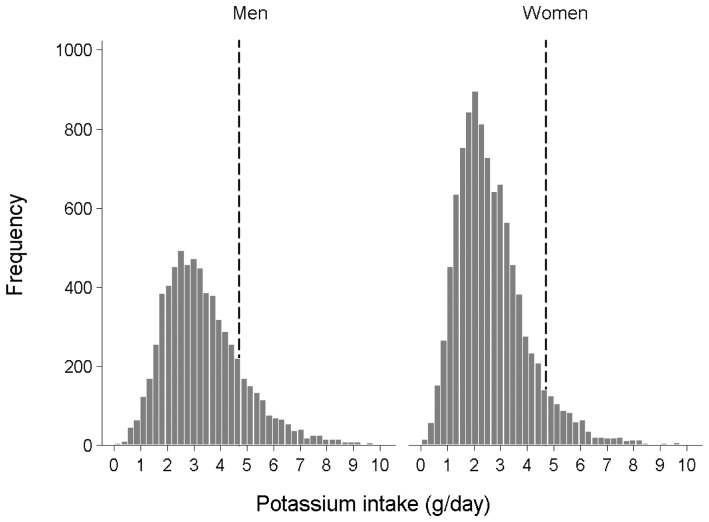
Different distribution of potassium intake according to sex. A histogram of potassium intake and numbers of participants is presented in men and women. Dashed lines reveal 4.7 g/day of potassium amount, which has been recognized as Adequate Intake of potassium.

**Figure 2:**
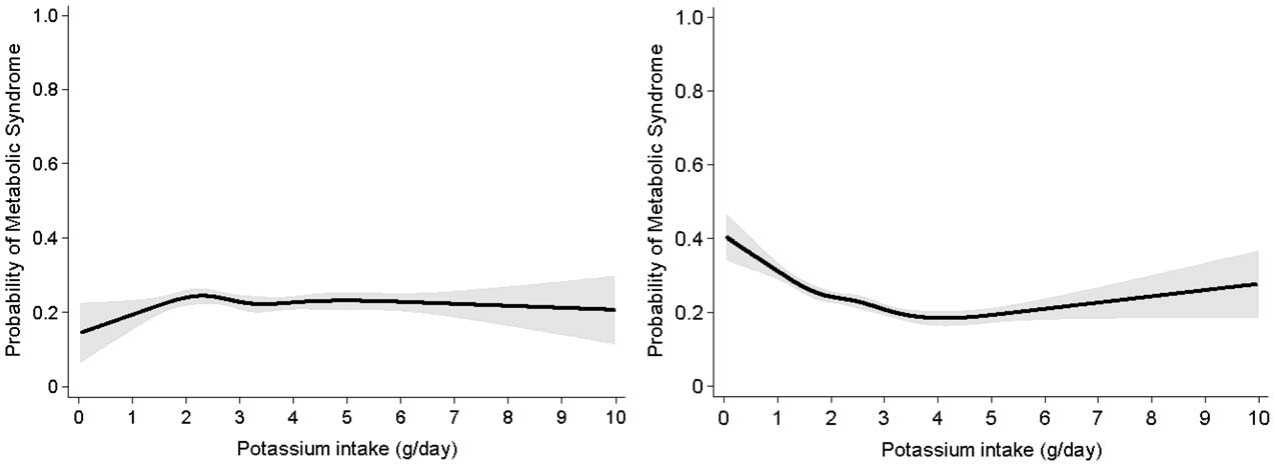
Unadjusted cubic spline of the relationship between potassium intake and metabolic syndrome according to sex. Probabilities of metabolic syndrome are presented in y axis in men and women. Gray shadow means 95% confidence interval.

**Figure 3:**
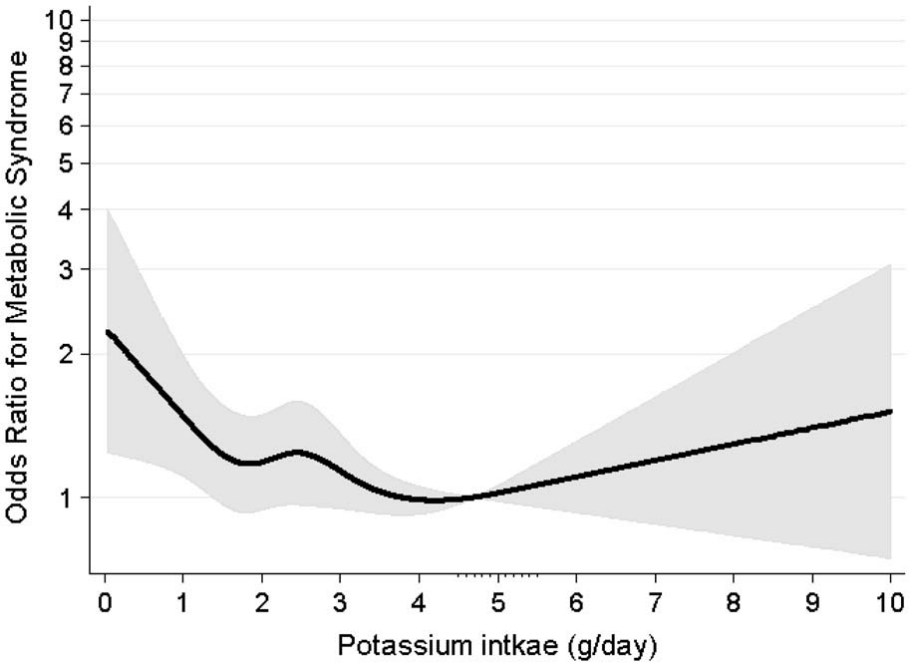
Adjusted cubic spline of the relationship between potassium intake and metabolic syndrome. The adjusted model includes age, body mass index, alcohol intake, education, income, frequency of fruit intake, energy intake and carbohydrate energy intake. Odds ratios for metabolic syndrome are presented in y axis as log scale. Gray shadow means 95% confidence interval.

Figure 1 represents the distribution of potassium intake according to gender. The proportion of individuals who consumed more than the Adequate Intake of potassium (4.7 g/day) was significantly lower among women (8.1%) than among men (17.3%). Figure 2 describes the relationship between potassium and the probability of MetS. Among men, there was no significant association between potassium intake and MetS risk. When the Adequate Intake level of 4.7 g/day was considered as the reference value, we found that women who consumed less than 4.7 g of potassium per day had an 11% of MetS risk reduction for every 1 g/day increase in potassium consumption (adjusted OR, 0.89; 95% CI, 0.82–0.96; *P = *0.004). On the other hands, in women consumed more than 4.7 g/day of potassium, the dietary potassium increment did not impact MetS risk (unadjusted OR, 1.09; 95% CI, 0.94–1.27; *P = *0.271). Figure 3 displays the restricted multivariable cubic spline plot that graphically describes the independent association between potassium intake and the risk for MetS among women. The curves were adjusted by age, BMI, alcohol intake, degree of education, income, frequency of fruit intake, energy intake, and carbohydrate energy intake. The curve shows that MetS risk declines sharply at a potassium consumption level of 4.7 g/day.

Then, women were also segmented into 6 potassium groups as follows: <1.5 g/day, 1.5–2.5 g/day, 2.5–3.5 g/day, 3.5–4.5 g/day (reference group), 4.5–5.5 g/day and ≥5.5 g/day. Table 2 compares demographic characteristics of 6 potassium group. In subjects consuming less than the reference amount of potassium, the prevalence of MetS became significantly higher as their potassium intake decreased. This was similar to the observed trend for the prevalence of diabetes and hypertension. The levels of HOMA-IR decreased as dietary potassium intake increased among those subjects. In addition, women with lower potassium intakes had a tendency to be older, smoke more, and were more likely to live in rural areas and be less educated. Moreover, they were physically inactive, poorer, and consumed fewer vegetables or fruit. The carbohydrate energy ratio was inversely correlated with potassium intake among women.

**Table 2:** Demographic characteristics of women according to potassium intake.

Variables	<1.5 g/dayn = 1697	1.5–2.5 g/dayn = 3513	2.5–3.5 g/dayn = 2559	3.5–4.5 g/dayn = 1235	4.5–5.5 g/dayn = 496	≥5.5 g/dayn = 405	*P*
Age (years)	54.5±19.1	48.7±16.6	47.4±15.0	47.5±13.9	47.1±13.3	48.5±13.2	<0.001
BMI (kg/m^2^)	23.6±3.7	23.4±3.5	23.4±3.3	23.3±3.2	23.0±3.0	23.4±3.27	0.016
Potassium intake (g/day)	1.11±0.30	2.00±0.28	2.97±0.28	3.92±0.29	4.94±0.29	6.62±1.05	<0.001
Potassium/energy	1.18±0.44	1.49±0.44	1.77±0.46	2.04±0.60	2.27±0.59	2.77±1.02	<0.001
Diabetes (%)	12.3	8.4	7.8	7.7	6.1	10.12	<0.001
Hypertension (%)	37.6	28.6	24.6	24.3	19.6	25.2	<0.001
Menopause (%)	60.0	45.4	41.5	42.8	42.8	48.0	<0.001
HRT (%)	8.1	14.6	18.4	21.9	27.6	22.7	<0.001
MS (%)	29.9	24.6	21.1	18.1	18.4	23.2	<0.001
Number of MS components	1.79±1.36	1.53±1.36	1.43±1.29	1.38±1.24	1.29±1.26	1.50±1.34	<0.001
High blood pressure (%)	45.0	38.3	34.9	36.3	32.3	33.1	<0.001
Hyperglycemia (%)	14.0	10.2	9.7	9.1	7.3	12.8	<0.001
Central obesity (%)	48.8	43.6	41.8	42.9	36.1	46.7	<0.001
Low HDL (%)	42.9	38.5	37.3	32.8	32.9	37.5	<0.001
Hypertriglyceridemia (%)	27.8	22.5	19.5	16.8	20.4	20.3	<0.001
HOMA-IR	2.56±1.59	2.42±1.71	2.39±1.45	2.37±1.98	2.26±1.39	2.27±1.14	<0.001
Smoking (%): Never	84.8	88.9	90.8	90.2	90.5	89.6	<0.001
Ex-smoker	1.6	1.6	1.7	2.3	1.6	2.7	
Current smoker	13.7	9.6	7.5	7.5	7.9	7.7	
Alcohol (%): <1/month	66.7	61.1	60.7	60.7	60.2	62.1	0.006
1/mon∼1/week	25	29.9	30.4	31.1	30.6	30.9	
≥2/week	8.2	9	8.9	8.2	9.1	6.9	
Exercise (%)	23.3	25.5	25.3	28.3	29.2	33.8	<0.001
Education (%): <middle	52.3	34.7	26.9	24.2	23.5	20.3	<0.001
Middle school	8.1	10.7	9.7	11.9	10.8	15.4	
High school	26.1	32.5	35.3	33.2	35.3	39.1	
≥University	13.6	22.2	28.2	30.8	30.4	25.3	
Income (%): 1Q	32	25.7	21.5	20.6	21.1	17.2	<0.001
2Q	25.2	26.2	24.6	23.1	20.7	22.7	
3Q	22.3	24.7	26.3	28.1	30.7	26.2	
4Q	20.6	23.4	27.6	28.2	27.6	33.9	
Urban residence (%)	39.4	42.8	44.7	44.6	46.7	44.2	<0.001
Vegetable intake (%)	30.9	38.6	41.0	43.5	47.9	47.5	<0.001
Fruit intake (%)	4.1	6.6	8.8	10.9	12.2	13.2	<0.001
Fiber (g/day)	3.0±1.6	5.2±2.2	7.4±2.9	9.9±3.9	12.8±5.3	18.9±10.1	<0.001
Energy intake (Kcal/day)	1011.8±357.5	1432.5±384.4	1774.0±451.3	2052.9±533.8	2316.7±603.4	2636.6±847.6	<0.001
CHO energy ratio (%)	73.9±11.9	70.6±11.4	69.2±11.6	69.0±11.7	69.7±12.0	71.4±13.3	<0.001

Data are presented with survey means ± SD for continuous variables and percentages for categorical variables. *P* values were based on the one-way analysis of variance test and χ^2^ test.

Abbreviations: BMI, Body mass index; MS, metabolic syndrome; HDL, high density lipoprotein; CHO, carbohydrate.

Table 3 summarizes the association between potassium intake and MetS in women. Women with lower potassium intakes had an increased risk for MetS in unadjusted state (1.5–2.5 g/day; OR, 1.35; 95% CI, 1.07–1.71, *P = *0.001; <1.5 g/day; OR, 1.86; 95% CI, 1.52–2.28; *P*<0.001). Even after considering the possible confounding factors, lower potassium intake was independently associated with a higher risk of MetS (1.5–2.5 g/day; OR, 1.29; 95% CI, 1.02–1.63; *P = *0.035; <1.5 g/day; OR, 1.40; 95% CI, 1.06–1.85; *P = *0.017). The relationship between potassium and MetS risk differed according to menstruation status. Among premenopausal women, the relationship was only significant in the univariate analysis and lost its significance after adjustment for age and BMI. In contrast, among postmenopausal women, the relationship revealed independent significance even after complete adjustment.

**Table 3:** Association between potassium intake and MS prevalence.

	OR (95% CI) according to potassium intake (g/day)					
Outcomes	<1.5 n = 1697	1.5–2.5 n = 3513	2.5–3.5 n = 2559	3.5–4.5 n = 1235	4.5–5.5 n = 496	≥5.5 n = 305
**Overall**						
Unadjusted	1.86 (1.52–2.28)	1.37 (1.14–1.66)	1.22 (1.00–1.49)	1 (ref.)	0.98 (0.70–1.36)	1.44 (1.01–2.04)
Model I	1.35 (1.07–1.71)	1.33 (1.07–1.65)	1.25 (1.00–1.56)	1 (ref.)	1.17 (0.82–1.67)	1.45 (0.99–2.12)
Model II	1.40 (1.06–1.85)	1.29 (1.02–1.63)	1.25 (0.98–1.57)	1 (ref.)	1.15 (0.79–1.67)	1.36 (0.92–2.01)
**Premenopause**						
Unadjusted	1.46 (1.01–2.10)	1.12 (0.81–1.54)	1.19 (0.86–1.65)	1 (ref.)	0.75 (0.86–1.65)	1.59 (0.92–2.76)
Model I	1.44 (0.90–2.30)	1.17 (0.80–1.70)	1.14 (0.78–1.67)	1 (ref.)	0.98 (0.51–1.87)	1.62 (0.91–2.90)
Model II	1.62 (0.95–2.75)	1.22 (0.82–1.81)	1.21 (0.82–1.79)	1 (ref.)	0.93 (0.47–1.85)	1.33 (0.73–2.41)
**Postmenopause**						
Unadjusted	1.74 (1.34–2.26)	1.63 (1.27–2.11)	1.37 (1.07–1.76)	1 (ref.)	1.16 (0.78–1.73)	1.19 (0.76–1.87)
Model I	1.34 (1.02–1.77)	1.46 (1.11–1.93)	1.32 (1.02–1.71)	1 (ref.)	1.32 (0.88–1.98)	1.30 (0.80–2.11)
Model II	1.30 (0.98–1.72)	1.38 (1.03–1.83)	1.28 (0.98–1.67)	1 (ref.)	1.29 (0.85–1.97)	1.34 (0.81–2.22)

Participants with potassium 3.5–4.5 g/day were reference group. Numbers are presented with percentages in parentheses in each potassium group. Odds ratios are given with 95% confidence interval in parentheses.

Model I: Adjusted for age, BMI.

Model II: Adjusted for Model I+alcohol intake, exercise, education, income, residential area, frequency of fruit intake, energy, and carbohydrate energy ratio.

Abbreviations: MS, metabolic syndrome; BMI, body mass index; OR, odds ratio; CI, confidence interval.

Through univariate analyses, we found that lower potassium intake was a significant determinant for all the 5 components of MetS. In the multivariate analysis, only hypertriglyceridemia was independently associated lower potassium intake. Detailed data were shown in the Table 4.

**Table 4:** Association between potassium intake and MetS components.

	OR (95% CI) according to potassium intake (g/day)					
Outcomes	<1.5 n = 1697	1.5–2.5 n = 3513	2.5–3.5 n = 2559	3.5–4.5 n = 1235	4.5–5.5 n = 496	≥5.5 n = 305
**High BP**						
Unadjusted	1.35 (1.13–1.61)	1.05 (0.90–1.22)	0.96 (0.81–1.13)	1 (ref.)	0.81 (0.62–1.06)	0.89 (0.68–1.17)
Model I	1.02 (0.83–1.24)	1.01 (0.86–1.18)	0.96 (0.80–1.14)	1 (ref.)	0.84 (0.64–1.10)	0.80 (0.59–1.07)
Model II	1.00 (0.78–1.28)	1.00 (0.84–1.19)	0.97 (0.81–1.16)	1 (ref.)	0.85 (0.64–1.13)	0.76 (0.56–1.04)
**Hyperglycemia**						
Unadjusted	1.69 (1.28–2.21)	1.04 (0.80–1.36)	1.03 (0.78–1.36)	1 (ref.)	0.65 (0.41–1.02)	1.54 (0.97–2.44)
Model I	1.07 (0.80–1.42)	0.89 (0.67–1.19)	1.00 (0.74–1.35)	1 (ref.)	0.71 (0.44–1.14)	1.51 (0.93–2.45)
Model II	0.96 (0.67–1.37)	0.81 (0.59–1.10)	0.94 (0.69–1.29)	1 (ref.)	0.73 (0.45–1.17)	1.68 (1.00–2.81)
**Central obesity**						
Unadjusted	1.27 (1.07–1.51)	1.02 (0.88–1.18)	0.97 (0.88–1.13)	1 (ref.)	0.82 (0.63–1.06)	1.29 (0.97–1.72)
Model I	1.05 (0.80–1.39)	1.02 (0.82–1.29)	0.92 (0.71–1.19)	1 (ref.)	0.85 (0.60–1.21)	1.32 (0.90–1.94)
Model II	1.04 (0.75–1.44)	1.00 (0.77–1.30)	0.91 (0.70–1.20)	1 (ref.)	0.82 (0.57–1.17)	1.19 (0.79–1.80)
**Low HDL-C**						
Unadjusted	1.44 (1.20–1.71)	1.17 (1.00–1.37)	1.24 (1.05–1.45)	1 (ref.)	0.97 (0.75–1.26)	1.22 (0.91–1.65)
Model I	1.27 (1.06–1.53)	1.16 (0.99–1.37)	1.25 (0.78–1.34)	1 (ref.)	1.02 (0.78–1.34)	1.18 (0.87–1.59)
Model II	1.19 (0.96–1.47)	1.11 (0.92–1.32)	1.21 (0.99–1.44)	1 (ref.)	1.01 (0.77–1.33)	1.10 (0.81–1.51)
**Hypertriglyceridemia**						
Unadjusted	1.80 (1.44–2.25)	1.30 (1.06–1.60)	1.19 (0.97–1.46)	1 (ref.)	1.09 (0.79–1.51)	1.07 (0.76–1.50)
Model I	1.48 (1.16–1.89)	1.28 (1.03–1.59)	1.20 (0.97–1.49)	1 (ref.)	1.20 (0.86–1.67)	1.02 (0.72–1.44)
Model II	1.49 (1.13–1.97)	1.22 (0.97–1.54)	1.20 (0.96–1.49)	1 (ref.)	1.18 (0.84–1.67)	0.99 (0.68–1.43)

Participants with potassium 3.5–4.5 g/day were reference group. Numbers are presented with percentages in parentheses in each potassium group. Odds ratios are given with 95% confidence interval in parentheses.

Model I: Adjusted for age, BMI.

Model II: Adjusted for Model I+alcohol intake, exercise, education, income, residential area, frequency of fruit intake, energy, and carbohydrate energy ratio.

Abbreviations: MetS: metabolic syndrome; BP, blood pressure; HDL-C, high-density lipoprotein cholesterol; BMI, body mass index; OR, odds ratio; CI, confidence interval.

### Potassium Intake and IR

Women who consumed less than 4.7 g of potassium per day had an approximately 10% IR risk reduction for every 1 g/day increase in potassium intake (adjusted OR, 0.90; 95% CI, 0.82–0.99, *P* = 0.026). In other words, women consuming less than 4.7 g of potassium per day had approximately 11% increase in MetS risk for every 1 g reduction in daily potassium intake. In women consuming more than 4.7 g/day of potassium, an increase in dietary potassium intake did not impact IR (unadjusted OR, 1.07; 95% CI, 0.92–1.24, *P* = 0.368). The continuous relationship between potassium intake and IR was also explored, and it appeared similar to the result for MetS even after adjustment for age, BMI, alcohol intake, physical activity, education level, income, energy intake and carbohydrate energy intake (data are not shown).

Table 5 summarizes the categorical association between potassium intake and IR. Lower potassium intake was associated with an increased risk of IR by multivariate analysis (<1.5 g/day; OR, 1.36; 95% CI, 1.05–1.76; *P* = 0.021), compared with the reference group.

**Table 5:** Association between potassium intake and insulin resistance.

	OR (95% CI) according to potassium intake (g/day)					
Outcomes	<1.5n = 1681	1.5–2.5n = 3488	2.5–3.5n = 2542	3.5–4.5n = 1229	4.5–5.5n = 491	≥5.5n = 405
**Overall**						
Unadjusted	1.60 (1.31–1.95)	1.16 (0.96–1.39)	1.21 (1.00–1.47)	1 (ref.)	0.74 (0.57–0.98)	1.17 (0.84–1.63)
Model I	1.49 (1.20–1.85)	1.14 (0.94–1.40)	1.22 (0.99–1.50)	1 (ref.)	0.81 (0.61–1.06)	1.13 (0.80–1.60)
Model II	1.36 (1.05–1.76)	1.07 (0.86–1.34)	1.21 (0.98–1.50)	1 (ref.)	0.85 (0.63–1.13)	1.24 (0.87–1.76)

Participants with potassium 3.5–4.5 g/day were reference group. Numbers are presented with percentages in parentheses in each potassium group. Odds ratios are given with 95% confidence interval in parentheses.

Model I: Adjusted for age, BMI.

Model II: Adjusted for Model I+alcohol intake, exercise, education, income, residential area, frequency of fruit intake, energy, and carbohydrate energy ratio.

Abbreviations: IR, insulin resistance; BMI, body mass index; OR, odds ratio; CI, confidence interval.

## Discussion

From this cross-sectional study, the relationship between dietary potassium and MetS was observed to differ according to gender. Moreover, higher dietary potassium intake was clearly shown to be an important determinant of MetS/IR risk, after multivariate adjustment including fruit and vegetable consumption among women. The relationship between potassium intake and MetS was more prominent among postmenopausal women than among those in the premenopausal state. Women who consumed less than the Adequate Intake level of potassium showed an 11% increase in MetS risk for every 1 g reduction in daily potassium intake. To the best of our knowledge, no previous study has established a clear relationship between potassium intake and MetS risk. Such observations are of clinical importance because most clinicians regard a higher potassium intake as cardiovascular protector related only to blood pressure [5], [22]. The described multivariate models revealed that potassium intake is not a simple derivation of fruit or vegetable intake, but is an independent risk factor for MetS. Furthermore, elevated potassium intake has an influence on lowering IR after adjusting confounding factors, including blood pressure.

The associations between potassium and glucose intolerance have been investigated in the areas of diabetes incidence and thiazide-induced hyperglycemia. Previous experimental studies demonstrated that potassium depletion causes glucose intolerance, which is associated with impaired insulin secretion [23]. Other prospective cohort studies have suggested that hypokalemia or lower potassium intake is related to an elevated incidence of type 2 diabetes risk among African-Americans [8], [24], and among women [25]. Moreover, a moderate potassium depletion, which did not induce frank hypokalemia, was proven to decrease plasma insulin concentrations and to induce resistance to insulin action. This effect was reversed by potassium supplementation [26]. In the case of thiazide induced glucose intolerance, further evidence supporting the role of hypokalemia in glucose metabolism can be derived from previous randomized controlled trials and meta-analyses [9], [10], [27], [28]. Although, some debates remain unsolved [29], a role for potassium deficiency in glucose intolerance seems reasonable [30]. Given the evidence above, there is only limited evidence suggesting that potassium intake has an effect on the prevalence of MetS, which is another representative disease territory for IR and an important cardiovascular risk factor. Until recently, research has focused only on the impact of thiazide induced hypokalemia on MetS [16], [31]. Therefore, the association between potassium deficiency and an incremental metabolic risk within the general population, as revealed in this study, is both novel and meaningful.

In the current study, the effect of potassium on MetS was proven only in women. Such a result might be due to the distribution of potassium intake across genders. As shown in a previous report [32], the current data also showed that potassium intake among women was generally lower than men. Approximately 92% of the women included in this study consumed less than the Adequate Intake level (4.7 g/day) of potassium, whereas this value was 82.7% among men. In addition, the proportion of subjects who consumed less than the daily minimal requirement of potassium, considered to be 1.6 g/day [33], was much higher among women (21.2%) than among men (9.2%). Therefore, it was assumed that women tended to consume less potassium than men in proportions to both the Adequate Intake level and the minimal requirement level. However, to clarify the precise mechanisms responsible for the gender-specific associations between potassium and MetS risk, further well-designed investigations are warranted**.**

Generally, women’s risk for cardiovascular diseases including MetS rapidly increases after menopause [14], [34], [35]. Although controversy remains regarding whether or not menopause itself increases cardiovascular risk independent of normal aging, estrogen deficiency and the progressive shift toward androgen dominance after menopause are suggested to influence cardiovascular risk [35], [36]. Interestingly, the study showed that nearly half of the women were menopausal and the pathologic role of potassium in MetS/IR was more prominent among them. The data suggest that lower potassium intake may affect development of MetS, particularly among postmenopausal women.

One of the strength of the present study is that it involved large, nation-wide, population-based sampling and also involved the collection of extensive data on potential confounders. All covariates were reliable and standardized through the use of a uniform questionnaire and surveillance protocol. Nevertheless, several limitations deserve mentioning. First, potassium intake may have been underestimated by the use of the 24-h dietary recall method, instead of a 24-h urinary collection. Indeed, correctly performed 24-h urinary collection considered the standard method for estimating daily potassium intake. However, urine collection over 24 h is usually difficult, inconvenient, and cumbersome particularly for women. Therefore, a large portion of the subjects cannot accurately collect the specimens. The 24-h recall method is a useful tool that can be applied to population-based researches, such as the present study [37], [38]. In addition, the correlation between dietary potassium intake and urinary potassium content is relatively high [21], [39], [40]. Although, the 24-h dietary recall method has its limitations, it is particularly useful in large scale studies. Second, because of the cross-sectional design, some factors, which may influence the observed associations, could not be considered. For example, dietary magnesium and serum ferritin levels may have an effect on MetS [41], [42]; however, neither of them was considered in this investigation. Another important factor that could not be considered in the present study was the subject’s medication history, including the use of thiazide or other diuretics, renin-angiotensin system blockade agents, or potassium-containing dietary supplements. Such medications may affect total body potassium amounts, in addition to dietary potassium intake. Third, serum potassium levels were not checked in this study and the role of hypokalemia in MetS risk could not be proven. Previous studies have reported that hypokalemia is associated with new onset diabetes and cardiovascular morbidity [7], [24]; however, the present study could not prove an impact of hypokalemia on MetS. In fact, dietary potassium is not directly related to serum potassium because serum potassium concentration is highly regulated to maintain intracellular homeostasis. Although unchecked serum potassium is one of the study’s limitations, hypokalemia is not believed to be a sensitive indicator of adequate potassium intake relative to the alleviation of metabolic risk. Moreover, the results could not prove a precise causal relationship between potassium intake and the risk of MetS because of the study’s cross-sectional design.

In conclusion, higher dietary potassium intake was associated with reduced MetS risk and decreased IR among women. The beneficial effect of potassium consumption was found to be independent of blood pressure or fruit or vegetable intake. This study is meaningful because it is the first to report relationship between potassium intake and MetS within the general population. Furthermore, dietary potassium intake can be suggested as a new, modifiable dietary factor to ameliorate MetS risk, particularly among women. Further investigation is warranted to determine whether or not potassium supplementation is effective in reducing MetS and IR, and, consequently, cardiovascular outcomes.
